# Highly Efficient and Stable MAPbI_3_ Perovskite Solar Cell Induced by Regulated Nucleation and Ostwald Recrystallization

**DOI:** 10.3390/ma11050778

**Published:** 2018-05-11

**Authors:** Zhen Huang, Duofa Wang, Song Wang, Tianjin Zhang

**Affiliations:** 1 Hubei Collaborative Innovation Center for Advanced Organic Chemical Materials, Ministry of Education Key Laboratory of Green Preparation and Application for Materials, Hubei Provincial Key Laboratory of Polymers, Department of Materials Science and Engineering, Hubei University, Wuhan 430062, Hubei, China; 15072474783@163.com; 2 Hubei Key Laboratory of Low Dimensional Optoelectronic Materials and Devices, Hubei University of Arts and Science, Xiangyang 441053, Hubei, China; wangsong1984@126.com

**Keywords:** perovskite solar cell, SnO_2_, low temperature, nucleation, antisolvent, recrystallization

## Abstract

Perovskite solar cells have attracted great attention in recent years, due to their high conversion efficiency and solution-processable fabrication. However, most of the solar cells with high efficiency in the literature are prepared employing TiO_2_ as electron transport material, which needs sintering at a temperature higher than 450 °C, and is not applicable to flexible device and low-cost fabrication. Herein, the MAPbI_3_ perovskite solar cells are fabricated at a low temperature of 150 °C with SnO_2_ as the electron transport layer. By dropping the antisolvent of ethyl acetate onto the perovskite precursor films during the spin coating process, compact MAPbI_3_ films without pinholes are obtained. The addition of ethyl acetate is found to play an important role in regulating the nucleation, which subsequently improves the compactness of the film. The quality of MAPbI_3_ films are further improved significantly through Ostwald recrystallization by optimizing the thermal treatment. The crystallinity is enhanced, the grain size is enlarged, and the defect density is reduced. Accordingly, the prepared MAPbI_3_ perovskite solar cell exhibits a record-high conversion efficiency, outstanding reproducibility, and stability, owing to the reduced electron recombination. The average and best efficiency reaches 19.2% and 20.3%, respectively. The device without encapsulation maintains 94% of the original efficiency after storage in ambient air for 600 h.

## 1. Introduction

Perovskite solar cells have attracted significant attention, due to their ability to exhibit efficient bipolar transport, tunable direct band gaps, high solar-to-electric power conversion efficiency (*PCE*), and solution process fabrication. The efficiency of these devices has also increased in recent years, from 3.8% to 22.1%, which makes it highly possible they could come into commercial mass production in the near future [1,2,3,4]. The typical perovskite solar cell is in the structure of fluorine-doped SnO_2_ (FTO) glass/electron transport layer (ETL)/perovskite/hole transport layer (HTL)/anode. Up to now, the most successful ETL is TiO_2_, and most of the highly efficient perovskite solar cells are TiO_2_ based [5,6]. Although TiO_2_ is the most widely used ETL for PSCs, the mobility (0.1–1 cm^2^ V^−1^ s^−1^) is even lower than that of CH_3_NH_3_PbI_3_ (MAPbI_3_, 20–30 cm^2^ V^−1^ s^−1^), making it not an ideal ETL material [7,8]. Moreover, the sintering temperatures for fabricating TiO_2_ ETL are very high (>450 °C), which is a barrier to the low-cost and stretchable device fabrication. Therefore, searching for other efficient ETLs is an important topic. Recently, ZnO [9], ZnAlO [10], SnO_2_ [11], and their composites with graphene have been reported as ETLs for perovskite solar cell [12]. Among the ETL materials above, SnO_2_ is very promising, because electron mobility is about 100–200 cm^2^ V^−1^ s^−1^, and it can also be fabricated at a temperature of 150 °C [13]. However, the *PCE* of these devices, including SnO_2_ based, is still much lower compared with those that are TiO_2_ based.

Very recently, it was reported that antisolvent engineering was an effective means to boost the *PCE* of solar cells [14,15]. The non-polar antisolvent does not dissolve perovskite precursor (PbI_2_ and CH_3_NH_3_I), but is quite miscible with DMF. It can remove the residual solvents of DMF rapidly, and boost the nucleation of the perovskite, which subsequently promotes the crystallization of the perovskite film [15]. Up to now, diethyl ether, chlorobenzene, and ethyl acetate (EA) have been selected as antisolvents in the literature [16,17,18]. Grätzel group employed a simple vacuum-flash solution processing method, and obtained shiny, smooth, and crystalline perovskite films and meso-structured TiO_2_/perovskite solar cells with a *PCE* of 19.6% [19]. Park employed diethyl ether as antisolvent during spin coating, which could extract the solvent quickly and promote the nucleation of the film, leading to an efficient TiO_2_/perovskite solar cell [15]. Different from the dropping method of addition of the antisolvent, Padture utilized an antisolvent bath method by dipping the substrate in the solution of diethyl ether, and achieved dense and crystalline perovskite film [20]. The antisolvent treatment is effective in promoting the nucleation and producing compact film, which is successful in obtaining efficient perovskite solar cells. However, the grain size of perovskite film prepared with antisolvent treatment is small compared with the traditional method [21], owing to the accelerated nucleation. Accordingly, there exists more grain boundaries in the film, which are recombination centers deteriorating the photovoltaic performance of the solar cell. Therefore, investigations on how to enlarge the grain size and improve the crystal quality of perovskite film is necessary to further enhance the *PCE*.

Here, MAPbI_3_ perovskite solar cells were fabricated at a low temperature of 150 °C, with SnO_2_ as ETL. By precise control of the nucleation with antisolvent technique and subsequent promotion of the crystallinity through Ostwald recrystallization, high quality perovskite films were obtained with uniform thickness, smooth surface, and high compactness. Consequently, the MAPbI_3_ perovskite solar cell exhibits a record-high *PCE* of 20.3%, with high stability and reproducibility.

## 2. Materials and Methods

Lead iodide (PbI_2_, 99.9%, from Yingkou Youxuan Trade Co., Ltd., Yingkou, Liaoning Province, China), CH_3_NH_3_I (MAI, ≥99.5%, from Xi’an Polymer Light Technology Corp, Xi’an, Shanxi Province, China), *N*,*N*-dimethylformamide (DMF, 99.8%), dimethyl sulfoxide (DMSO), 2,2′,7,7′-tetrakis [*N*,*N*-di(4-methoxyphenyl)amino]-9,9′-spirobifluorene (spiro-OMeTAD), and (6,6)-phenyl C61 butyric acid methyl ester (PCBM) were purchased from Sigma-Aldrich (St. Louis, MO, USA), and used as received. The SnO_2_ colloid precursor was from Alfa Aesar (Ward Hill, MA, USA, 15% in H_2_O colloidal dispersion).

The etched substrates of fluorine doped SnO_2_ (FTO) were cleaned with deionized water, acetone, isopropanol, and ethanol in an ultrasonic cleaner for 30 min. After being dried by the N_2_ flow, the substrates were put in an ultraviolet–ozone environment for 30 min to remove organic residues. The SnO_2_ colloid precursor from Alfa Aesar was diluted with water (1:3, volume ratio) to prepare the precursor. Then, it was spin-coated onto the FTO substrate with a speed of 4000 rpm for 30 s, and heated at 150 °C for 30 min. The perovskite film was deposited over SnO_2_ ETL by one-step spin coating at 1000 rpm for 10 s, followed by 3500 rpm for 20 s, with the precursor composed of PbI_2_, MAI, and DMSO (1.6 M for each) in 1 mL DMF. Ethyl acetate was dropped onto the perovskite film at the last 10th second during the spin coating. As soon as the spin coating was finished, the sample was moved to a hotplate and annealed for 10 min at a temperature 100–140 °C. The spiro-OMeTAD in chlorobenzene (72.3 mg/mL) solution with Li-TFSI and TBP additive was spin-coated onto the top of annealed perovskite layer to prepare hole transport materials. Finally, Au electrode with a thickness of 80 nm was deposited by thermal evaporation.

Photocurrent density–voltage (*J*–*V*) curves were acquired using a Keithley 2400 (Keithley Instruments, Inc., Cleveland, OH, USA) source meter under AM 1.5 G one-sun illumination provided by a solar simulator 91192-1000 (Newport Corporation-Oriel Instruments, Mountain View , CA, USA). Each curve was generated using 60 data points. The active area of the cell is 0.1 cm^2^ and the scan rate is 40 mV/S. The incident photon to current efficiency (*IPCE*) was obtained using CIMPS-S optical system. The morphologies of the samples were investigated with a field emission scanning electron microscope (SEM, JSM-6700F, Japan Electronics Corporation, Tokyo, Honshu, Japan). The crystal structures of the MAPbI_3_ films were assessed by X-ray diffraction (XRD, D8 advance, Bruker, Karlsruhe, Germany) with Cu Ka radiation (λ = 1.54178 Å). Optical absorption spectra were obtained with a UV-3600 spectrophotometer (Shimadzu, Kyoto, Japan) over the range from 300 to 900 nm. The defect density in perovskite film was obtained by measuring the dark current density–voltage (*J*–*V*) of the electron-only device with a glass/ITO/PCBM/perovskite/PCBM/Au architecture. In this device, the PCBM layer was deposited by spin coating PCBM solution in chlorobenzene. The perovskite layer and Au electrode were prepared as the same process as the solar cell fabrication.

## 3. Results and Discussion

### 3.1. Regulated Nucleation by Anti-Solvent Engineering

**Figure 1:**
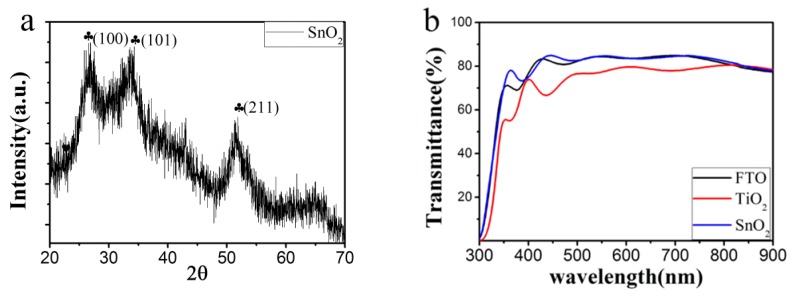
(**a**) X-ray diffraction pattern of SnO_2_ film; (**b**) Transmittance spectra of bare fluorine-doped SnO_2_ (FTO) glass and TiO_2_, SnO_2_ films on FTO glass.

The crystal structure of the SnO_2_ ETL was characterized by X-ray diffraction, performed on the film deposited on glass without FTO conducting layer. As shown in Figure 1a, all the diffraction peaks are indexable to the tetragonal SnO_2_ structure (space group P42/mnm), indicating the formation of pure SnO_2_ crystals. Transmission spectrum characterization in Figure 1b reveals that the SnO_2_ ETL is extremely transparent. The transmittance of the SnO_2_ film deposited on FTO glass is comparable with bare FTO glass, much better than that of the conventionally used TiO_2_ ETL. The high transmittance of SnO_2_ minimizes the loss of photons before reaching the perovskite active layer, and improves the light absorption in the active layer when the sample is back incident.

**Figure 2:**
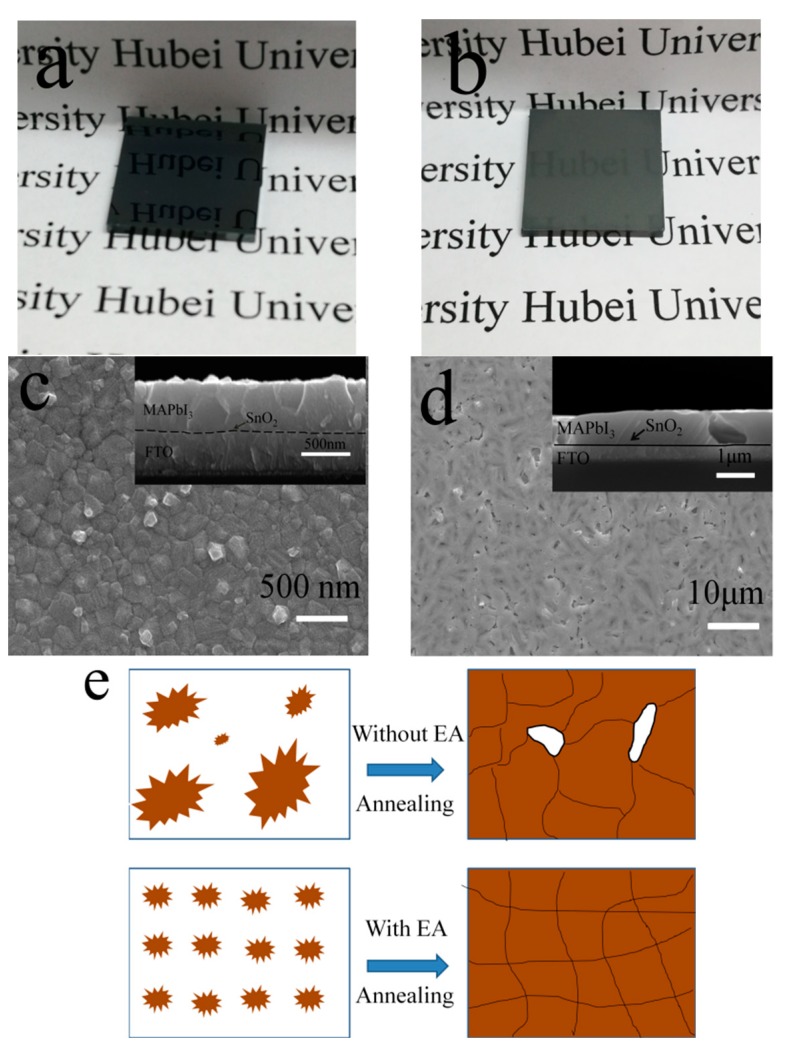
Photograph of MAPbI_3_ perovskite films fabricated with (**a**) and without (**b**) ethyl acetate (EA) treatment; Top-view SEM images of MAPbI_3_ films with (**c**) and without (**d**) EA treatment, insets are the corresponding cross-sectional SEM images; (**e**) Schematic illustration of nucleation and crystallization of perovskite film without/with EA treatment.

The perovskite film was prepared by spin coating MAPbI_3_ precursor containing DMSO onto the top of the compact SnO_2_ ETL, as described in the experimental section. The antisolvent of EA was dropped onto the perovskite film at the last tenth of a second during the high-speed spinning stage. We observed that the application of EA treatment influenced considerably the formation of perovskite film. A smooth and transparent film was formed quickly after the dropping of EA, as shown in the video (Supplementary Materials, Video S1). This transparent film is indicative of the formation of a Lewis adduct with the composition of DMSO–PbI_2_–CH_3_NH_3_I, as revealed in previous reports by Fourier transform infrared spectrometer and XRD [15]. As soon as the spin coating step was done, the sample was placed on a hotplate, and annealed at a temperature of 100 °C. It was seen that a dark brown perovskite film was formed after several minutes annealing. The surface of the film is ultra-smooth, and specular reflection is obviously observed, as shown in Figure 2a and in Video S1 in the Supporting Information. Whereas, the as-deposited film without EA treatment is rough, gray, and semitransparent, as shown in Figure 2b and in Video S2 in the Supporting Information. Moreover, the time consumed to form perovskite film is much longer in the case without EA treatment.

Scanning electron microscopy (SEM) was employed to investigate the effect of EA treatment on the microstructure of the perovskite film. As shown in Figure 2c,d, the difference between the MAPbI_3_ perovskite films without and with EA treatment is stark. The conventional one-step solution deposition without EA treatment induces typical branchlike crystals, and the SnO_2_ ETL is not fully covered. Whereas, homogeneous perovskite film without pinholes is formed with the EA treatment, covering the SnO_2_ ETL completely. The cross-sectional SEM images in the insets reveals that the perovskite film with EA treatment is extremely uniform and highly dense, which is comparable to the film quality obtained by vapor deposition method [22]. Whereas, big voids exist in the sample without EA treatment and extend to the whole depth of perovskite layer (see inset of Figure 2d). The improved morphology of the perovskite film by the antisolvent EA is ascribed to the uniform nucleation and crystallization, as illustrated by the scheme in Figure 2e. The non-polar EA does not dissolve perovskite precursor (PbI_2_ and CH_3_NH_3_I) but is quite miscible with DMF. Therefore, it can remove the residual solvents of DMF rapidly and boost the nucleation of the perovskite. Subsequently, an abundance of nuclei is formed and uniformly distributed on the substrate, which grow into uniform grains and ultimately coalesce into a dense polycrystalline film under thermal annealing, as illustrated in Figure 2e. In the case without EA, nuclei on the substrate are formed randomly, and the number is comparably much less. The nuclei continue to grow controlled by Ostwald ripening under thermal annealing. In this case, gaps are easily formed between the grains.

**Figure 3:**
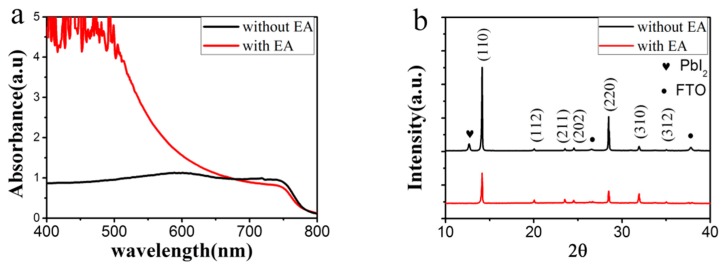
(**a**) UV-vis absorption spectra and (**b**) XRD pattern of the MAPbI_3_ perovskite without/with EA treatment. The heart and dot denote diffraction peaks corresponding to PbI_2_ and FTO films, respectively.

Ultraviolet–visible light (UV-vis) absorption spectra of the films with/without EA treatment are compared in Figure 3a. The absorption edges for both spectra are close to 780 nm, which is in agreement with literature [23]. The intensity of the absorption peak for the film with EA treatment is very strong. Contrarily, the intensity without EA treatment is rather weak, which is consistent with the photograph in Figure 2b that the sample is semitransparent. The inferior absorption for the untreated one is ascribed to the low compactness of the perovskite film, in which the FTO glass is not fully covered, as shown in Figure 2. The crystallinity of the MAPbI_3_ films is characterized by X-ray diffraction pattern and shown in Figure 3b. All the diffraction peaks corresponding to the orthorhombic MAPbI_3_ are indexed in the pattern. No obvious difference in the position of the diffraction peaks is observed for the MAPbI_3_ films with/without EA treatment. The full width at half maximum (FWHM) of the (110) diffraction peak of MAPbI_3_ film, without and with EA treatment, is 0.088 and 0.115, respectively. It reveals that the grain size of the non-treated sample is larger, which is consistent with SEM results. Moreover, an additional diffraction peak at 12.65° is observed in the XRD pattern of the non-treated sample, which corresponds to the (001) plane of PbI_2_. However, it is absent for the MAPbI_3_ film with EA treatment. This indicates that employment of antisolvent EA not only improves the compactness of the film, but also promotes the conversion of PbI_2_ to perovskite MAPbI_3_.

### 3.2. Improved Quality of Perovskite Film by Ostwald Recrystallization

**Figure 4:**
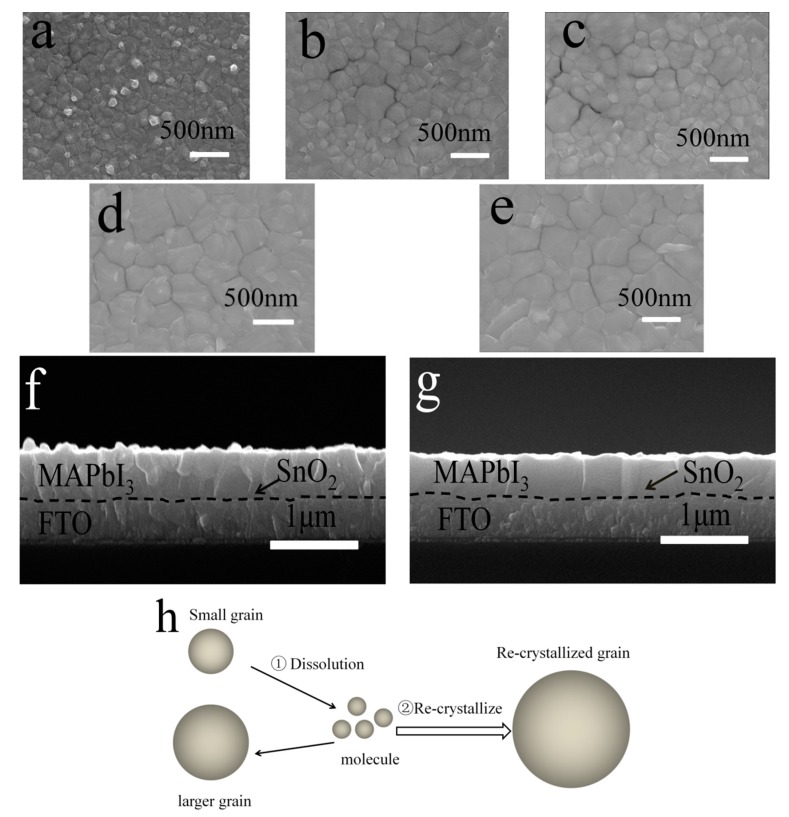
SEM images of perovskite films annealed (**a**) 100 °C; (**b**) 110 °C; (**c**) 120 °C; (**d**)130 °C; (**e**) 140 °C; (**f**) Cross-sectional SEM images of the perovskite films annealed at (**f**) 100 °C and (**g**) 130 °C; (**h**) Illustration of Ostwald recrystallization process.

In the literature regarding work on antisolvent assisted fabrication, the MAPbI_3_ perovskite films were mostly annealed at 100 °C, which was the optimized temperature for the traditional fabrication method. However, the nucleation and crystallization dynamics are different when antisolvent is employed. Therefore, we investigate the effect of annealing temperature on the morphology and crystallinity of the perovskite film, to improve the crystal quality of the perovskite film. Figure 4 is the SEM images of the perovskite film annealed at different temperatures. As shown, the crystallinity is significantly improved with the increase of temperature. The average grain size is increased from 150 to 500 nm when the temperature is increased from 100 to 130 °C. The surfaces of the films become smoother as well. Columnar crystals with heights reaching the whole thickness of the perovskite film are clearly observed, as shown in the cross-sectional SEM image (see Figure 4g). Further increasing the temperature to 140 °C leads to increased gaps between the grain boundaries instead. The enlarged grain size can be explained by the Ostwald recrystallization model, as illustrated in Figure 4h. The Ostwald ripening process is driven by the surface energy, and normally involves two coupled steps. The first step is the dissolution of small-sized crystals, because of the higher equilibrium vapor pressure (process ① in Figure 4h); the second step is the formation of large-sized crystals to decrease the surface energy (process ② in Figure 4h). At higher temperatures, more small-sized crystal are dissolved and recrystallized to form larger-size crystals, leading to improved film quality.

**Figure 5:**
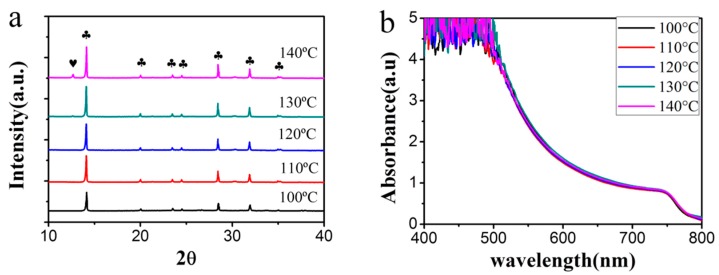
(**a**) XRD patterns and (**b**) UV-vis absorption spectra of the perovskite films annealed at different temperatures. The heart and club denote diffraction peaks corresponding to PbI_2_ and MAPbI_3_, respectively.

The XRD patters and UV–vis absorption spectra of the MAPbI_3_ films annealed at different temperatures are measured and shown in Figure 5. As seen, orthorhombic perovskite MAPbI_3_ films are formed at all temperatures. The intensity of diffraction peaks is increased and the full width at half maximum is decreased, with the increase of temperature, indicating the enhancement of the crystallinity. When the temperature reaches 140 °C, a diffraction peak corresponding to PbI_2_ at 12.65° is observed. It is ascribed to be induced by the decomposition of MAPbI_3_, since it only appears at the highest temperature. In the UV-vis absorption spectra, no distinct difference is observed among the samples prepared at different temperatures, indicating that all perovskite films show similar absorption efficiency.

### 3.3. Photovoltaic Properties of the Perovskite Solar Cell

**Figure 6:**
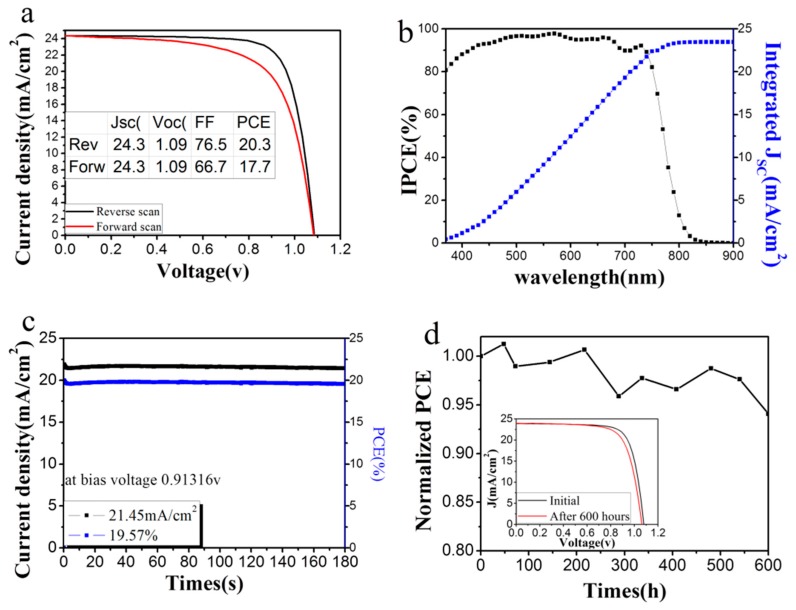
(**a**) *J*–*V*; (**b**) Incident photon to current efficiency (*IPCE*; black) and integrated short-circuit current density (blue) of the solar cell annealed at 130 °C; (**c**) The maximal steady-state power conversion efficiency (blue) and photocurrent output (black); (**d**) Stability data of cell stored in ambient air, inset is the *J*–*V* curves of the device, which are measured initially and after 600 h storage.

Perovskite solar cells with a structure of FTO/SnO_2_/MAPbI_3_/Spiro-OMeTAD/Au are fabricated, and the photovoltaic performance measured under AM 1.5 simulated solar illumination (100 mW/cm^2^). It is found that the perovskite solar cell prepared at 130 °C exhibits the highest *PCE*, which is consistent with the SEM and XRD results that it maintains the highest crystallinity and compactness. Figure 6a plots the *J*–*V* curve of the perovskite cell annealed at 130 °C. The *PCE* is 19.24 ± 1.06% (the best *PCE* reaches 20.3%) in the reverse scan, and 17.2 ± 0.81% (the best *PCE* is 18.01%) in the forward scan. Due to the relatively larger bandgap of MAPbI_3_ (1.55 eV) compared to FAPbI_3_ (1.48 eV), the *PCE* is slightly lower than the formanidinium lead trihalide (FAPbI_3_) solar cell reported by You group (20.5%) [24]. However, this is the record *PCE* value for MAPbI_3_ perovskite solar cell with SnO_2_ ETL, as far as we know. A summary of the perovskite solar cell based on SnO_2_ ETL with *PCE* higher than 15% in literature is shown in Table 1. In the typical incident photon-to-electron conversion efficiency (*IPCE*) plot in Figure 6b, rather high values are achieved, and the highest reaches 97% in the visible region. The integrated short-circuit current is 23.76 mA/cm^2^ and matches well with the *J*–*V* results. Moreover, the cell shows very stable output current density and *PCE* at the maximal power point, as shown in Figure 6c. It delivers stabilized current density of 21.45 mA/cm^2^ and *PCE* of 19.57% at 0.913V, which is comparable to the values obtained from the *J*–*V* scan. The stability measurement of the solar cells without encapsulation is performed by storing the device in the lab (30% humidity, under laboratory light illumination) and measuring the *PCE* every 60 h. As shown in Figure 6d, the solar cell exhibits high stability, the *Jsc* is nearly unchanged, the *Voc* decreased from 1.08 V to 1.06 V, the *FF* decreased from 76% to 72%, and the efficiency decreased from 19.57% to 18.35% (94% of the initial value) after 600 h.

**Figure 7:**
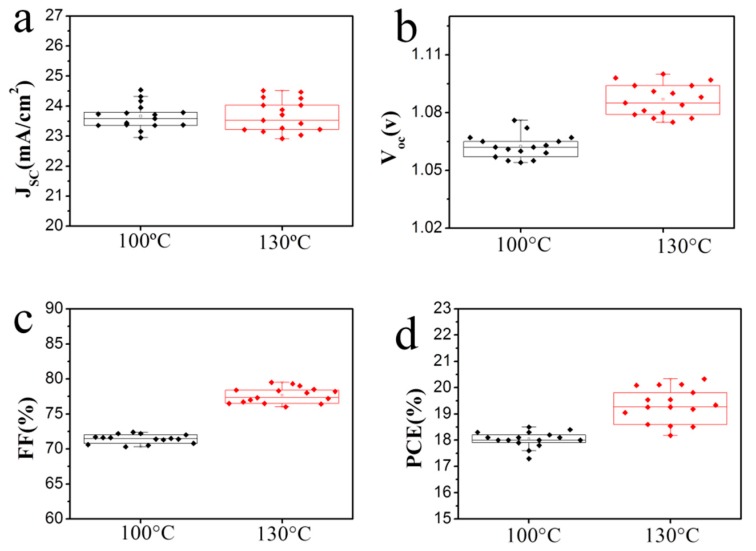
Statistical distribution of the photovoltaic parameters for the MAPbI_3_ solar cell anneal at 100 °C and 130 °C. (**a**) *Jsc*; (**b**) *Voc*; (**c**) fill factor; (**d**) *PCE*.

To investigate the effect of annealing temperature on the photovoltaic performance of the solar cell and to reveal the mechanism, we compared the photovoltaic characteristics and the distribution of the solar cells annealed at 100 and 130 °C. As shown in Figure 7a, the cells exhibit similar *Jsc*. It indicates that the two MAPbI_3_ films maintain similar light absorption efficiency since it is the most important factor affecting *Jsc*. This is consistent with the UV-vis results in Figure 5. In Figure 7b and Figure 7c, an obvious increase in *Voc* and *FF* is observed when the temperature is increased. It reveals that the recombination rate of electrons is lower in the cell of 130 °C, which leads to the higher *PCE*, as shown in Figure 7d. The lower recombination rate can be ascribed to the improved crystallinity by high temperature annealing. When the annealing temperature is increased to 130 °C, abundant grain boundaries disappear, and accordingly, the defect density is reduced, leading to the increased lifetime and decreased recombination rate of electrons.

**Figure 8:**
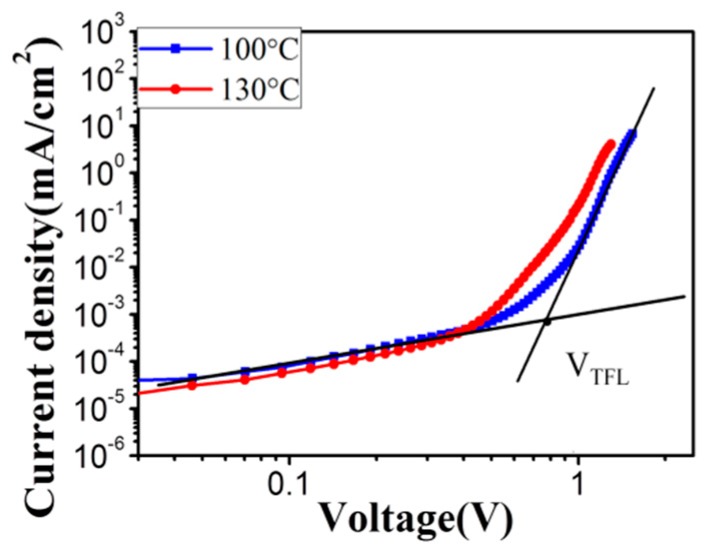
Dark *J*–*V* characteristics of the electron only device based on MAPbI_3_ perovskite film annealed at 100 °C and 130 °C.

To confirm the dependence of defect density on the annealing temperature, we fabricated an electron-only device with the glass/ITO/PCBM/perovskite/PCBM/Au architecture, and assessed the trap density in these devices. The dark *J–V* characteristics were measured to obtain the electron trap densities. Figure 8 shows the typical dark *J–V* characteristics of electron-only devices with the perovskite film annealed at 100 and 130 °C. The trap densities were estimated by the space charge–limited current (SCLC) model [40]. The linear region at low bias corresponds to the ohmic response. The current quickly increases when the bias voltage exceeds the kink point, demonstrating that the trap states are completely filled. The trap-state density can be determined by the trap-filled limit voltage ($V_{TEL}$) using equation $V_{TEL}=en_{t}L^{2}/2\varepsilon \varepsilon_{0}$, where L is the thickness of the perovskite film, ε is the relative dielectric constant of the perovskite (taken as the value 32 for MAPbI_3_ according to a previous report [41]), $\varepsilon_{0}$ is the vacuum permittivity, and $n_{t}$ is the trap density. The electron trap densities are calculated to be 9.8 × 10^15^ and 7.6 × 10^15^ cm^−3^ for the films annealed at 100 and 130 °C, respectively. It explains the difference of the photovoltaic performance very well, that the solar cell annealed at 130 °C exhibits higher *Voc* and *FF*, which is induced by the lower recombination rate of electrons.

## 4. Conclusions

In summary, MAPbI_3_ perovskite solar cells with SnO_2_ as ETL were fabricated at a low temperature of 150 °C. Compact perovskite film without pinholes is obtained by dropping the antisolvent of ethyl acetate onto the perovskite precursor films during the spin coating process. Because the antisolvent (ethyl acetate) does not dissolve perovskite precursor, but is quite miscible with DMF, the addition of antisolvent effectively extracts residual solvent (DMF), and accelerates the nucleation of MAPbI_3_ perovskite, which accordingly improves the compactness of the MAPbI_3_ films. The quality of MAPbI_3_ film was further improved significantly through Ostwald recrystallization process, by optimizing the thermal treatment. The crystallinity is enhanced, the grain size is increased three times, columnar crystals with heights reaching the whole thickness of the film are formed, and the defect density in MAPbI_3_ film is reduced from 9.8 × 10^15^ to 7.6 × 10^15^ cm^−3^ when the annealing temperature is increased from the widely performed temperature of 100 to 130 °C. Consequently, MAPbI_3_ perovskite solar cell with record-high conversion efficiency, and outstanding reproducibility and stability is achieved. The average and best efficiency reaches 19.2% and 20.3%, respectively. The device without encapsulation maintains 94% of the original efficiency after storage in ambient air for 600 h.

## Figures and Tables

**Table 1:** Summary of perovskite solar cell based on SnO_2_ ETL with efficiency higher than 15% in literature.

Electron Transport Layer	Perovskite	Efficiency (The Best)	Ref.
SnO_2_	(FAPbI_3_)_0.97_(MAPbBr_3_)_0.03_	20.5%	[24]
Nb/SnO_2_	MAPbI_3_	17.57	[25]
MgO/SnO_2_	MAPbI_3_	18.82%	[26]
mp-SnO_2_	MAPbI_3_	16.17%	[27]
C60/SnO_2_(invert)	MAPbI_3_	18.8%	[28]
SnO_2_/PCBM	MAPbI_3_	19.45%	[29]
SnO_2_	MAPbI_3_	15.07%	[30]
SnO_2_	(FAPbI_3_)_0.85_(MAPbBr_3_)_0.15_	18.4%	[31]
SnO_2_	(FAPbI_3_)_0.8_(MAPbBr_3_)_0.2_	20.8%	[32]
SnO_2_	MAPbI_3_	17.2%	[33]
SnO_2_	MAPbI_3_	18.32%	[34]
Li/SnO_2_	MAPbI_3_	18.2%	[35]
SnO_2_	MAPbI_3_	18.16%	[36]
SnO_2_	MAPbI_3_	18.77%	[37]
ZnO–SnO_2_	MAPbI_3_	15.2%	[38]
SnO_2_	FA_0.8_Cs_0.2_PbI_3_	19.57%	[39]
SnO_2_	MAPbI_3_	20.3%	Our work

## Supplementary Materials

The following are available online at https://zenodo.org/record/1246032#.WvfxYVKSCJ0, Video S1: fabrication process of the MAPbI_3_ film with antisolvent treatment, Video S2: fabrication process of the MAPbI_3_ film without antisolvent treatment.
